# Implementation contexts of a Tuberculosis Control Program in Brazilian prisons

**DOI:** 10.1590/S0034-8910.2015049005802

**Published:** 2015-09-23

**Authors:** Luisa Gonçalves Dutra de Oliveira, Sonia Natal, Luiz Antonio Bastos Camacho

**Affiliations:** I Departamento de Planejamento em Saúde. Instituto de Saúde Coletiva. Universidade Federal Fluminense. Niterói, RJ, Brasil; II Departamento de Saúde Pública. Universidade Federal de Santa Catarina. Florianópolis, SC, Brasil; III Departamento de Epidemiologia e Métodos Quantitativos em Saúde. Escola Nacional de Saúde Pública Sérgio Arouca. Fundação Oswaldo Cruz. Rio de Janeiro, RJ, Brasil

**Keywords:** Prisoners, Prisons, Tuberculosis, prevention & control, Program Evaluation, Qualitative Research

## Abstract

**OBJECTIVE:**

To analyze the influence from context characteristics in the control of tuberculosis in prisons, and the influence from the program implementation degrees in observed effects.

**METHODS:**

A multiple case study, with a qualitative approach, conducted in the prison systems of two Brazilian states in 2011 and 2012. Two prisons were analyzed in each state, and a prison hospital was analyzed in one of them. The data were submitted to a content analysis, which was based on external, political-organizational, implementation, and effect dimensions. Contextual factors and the ones in the program organization were correlated. The independent variable was the program implementation degree and the dependent one, the effects from the Tuberculosis Control Program in prisons.

**RESULTS:**

The context with the highest sociodemographic vulnerability, the highest incidence rate of tuberculosis, and the smallest amount of available resources were associated with the low implementation degree of the program. The results from tuberculosis treatment in the prison system were better where the program had already been partially implemented than in the case with low implementation degree in both cases.

**CONCLUSIONS:**

The implementation degree and its contexts – external and political-organizational dimensions – simultaneously contribute to the effects that are observed in the control of tuberculosis in analyzed prisons.

## INTRODUCTION

The actions aiming to control tuberculosis (TB) in the Brazilian prison system were standardized by Brazilian National Prison System Health Care Plan, in 2003, and by the recommendations from Brazilian National Tuberculosis Control Program, in 2010. The agreed objectives were: achieving a cure rate of at least 85.0%, in order to reduce transmission and new cases; and to reduce treatment abandonment to less than 5.0%, with better prognoses, and decreased risk of resistance to first-line drugs.

Each federated unit plans and executes their *Plano Operativo Estadual* (POE – State Operation Plans) for Prison Health Care. There may be different ways to organize and manage the actions. The integration among penitentiary and health care management bodies at federal, state, and municipal levels is fundamental for the development of actions with established purposes by professionals who are motivated and qualified to promote early diagnose and successful treatment.^17^ ^,^ ^a^ The Municipal Health Care Offices of cities housing prisons contribute to the conduction of complementary exams, more complex actions, and health care to inmates. Besides the biomedical approach, educational measures and measures to improve environmental conditions are required to control the disease in prisons.^b^

Funding of prison health care programs is shared by the Ministries of Justice and Health, with the participation from state prison and health care management bodies. Financial incentives to prison health care, as established in 2003, are destined to prisons with over 100 inmates and their own health care units with minimum teams.^c^

There is little knowledge on the factors that influence the implementation of actions by the Tuberculosis Control Program (TCP) in the prison system. This study is one of the steps of research “Avaliação da Implantação do PCT no Sistema Penitenciário Brasileiro” (Evaluation of TCP Implementation in the Brazilian Prison System). The first step, a evaluability study, found the existence of different organization models in the selected cases.^14^ In the second step, a multiple case study,^11^ ^,^ ^19^ the implementation degrees of the program were analyzed in five prisons of two State Prison Systems.^16^

This study aimed to analyze the influence from context characteristics in the control of tuberculosis in prisons, and the influence from the program implementation degrees in observed effects.

## METHODS

A multiple case study^10^ ^,^ ^11^ ^,^ ^19^ with a qualitative approach. The criteria for selecting cases were: existence of implemented TCP, with on-site outpatient or inpatient treatment, and existence of a large number of new TB cases registered. The selected cases, one of which with the highest TB incidence on the prison system and the other one with the biggest inmate population in the country, had different contexts and organization models for the program.^14^ The prisons considered were the ones with male inmates, due to the majority of men in the prison system and a higher prevalence of TB in both free and incarcerated men. The cases were the Prison Health Care Systems of two states; the analysis units, two prisons from each state. A prison hospital was included, in one of the cases, among the analysis units, where actions were centralized.

The dimensions used in the theoretical model of the program were: external, political-organizational, implementation *per se*, and effects. The external dimension, which corresponds to the state context, had sociodemographic vulnerability and political commitment as its sub-dimensions. The categories defined for the first one were the demographic aspects, the socioeconomic status of the population, TB incidence, and Aids prevalence, as factors which influence the population susceptibility to tuberculosis transmission and pathogenesis. Sub-dimension political commitment was determined by the logistic and material support to health care actions by the health care offices and prison administration offices.

The analyzed sub-dimensions of the political-organizational dimension were technical autonomy and political-financial autonomy, intersectorial actions, and management. In the first one, the categories management of POE (State Operation Plans) and use of financial incentives indicated the participation from managers and health care professionals in the planning and management of funds and actions from the program. The intersectorial actions, integration between health care and security areas, intended to ensure the execution of TB control actions in the prisons. In management, the TCP organization models in the state prison systems and the established flows for diagnosing, supervising, and treating the cases were considered.

Dimension implementation corresponded to the execution of TB control actions as standardized, and dimension effects considered the operational results: cure and lethality rates, treatment adhesion, and inmate satisfaction regarding health care.

The theoretical model of the program used the type of implementation analysis that was proposed by Champagne et al^7^ (Figure): (1) The external and political-organizational context determining factors may favor or hinder the implementation of TB control actions in prisons; (2) The program implementation degree influences the effects which are attributed to it; (3) The interaction among the dimensions may also influence observed results.

**Figure:**
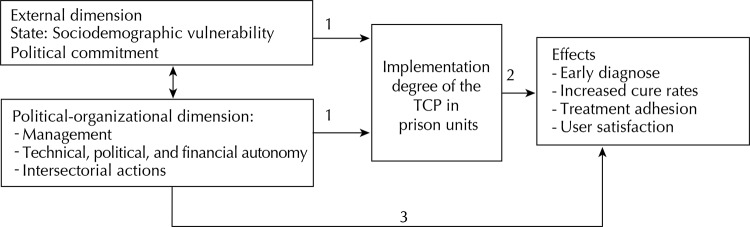
Theoretical Model of the Tuberculosis Control Program in the prison system. Brazil, 2012.

The previous step of this study outlined the degrees of TCP implementation in the several analysis units according to the results that were obtained for five categories: access; infrastructure; search of cases; diagnostics and supervision; and pharmaceutical treatment and assistance. Thresholds were established according to the ratios between total points in the categories and estimated scores. Partially implemented programs (from 51.0% to 75.0%) were found in three analysis units. Two analysis units were found to have low implementation degrees (from 26.0% to 50.0%).^16^

The implementation degree of the intervention was considered as the dependent variable, in order to analyze the influence from external and political-organizational dimensions in the implementation in both cases. The analysis of how the differences in implementation degrees made the effects vary had the posited effect as the dependent variable. The process through which the interaction between the intervention and its context influenced the effects was analyzed.

The primary data were obtained by semi-structured interviews and field observations, by the use of previously drafted scripts as well as audio and written field records. The director of health care actions and the health care directors in the two selected prisons were interviewed as representatives from the prison health care coordination, in case 1. The person responsible for the prison TCP and the director of the prison hospital for TB treatment were interviewed in case 2. In the five analysis units, 37 health care professionals (physicians, nurses, nursing assistants, dentist, and social assistant) were interviewed, as well as 14 guards who had been jointly selected with the chief of security (with priority to the ones who worked at the health care units), and 42 inmates on TB treatment.

The secondary data came from the program documents and regulations, prison health care legislation, POE, information provided by the The Brazilian Institute of Geography and Statistics (IBGE) and the Information System on Diseases of Compulsory Declaration (SINAN),^d^ the Penitentiary Information Integrated System,^e^ Management reports, and National Register of Health Facilities.^f^ The quantitative data were presented as absolute numbers and percentages.

A content analysis^3^ was used to analyze the qualitative data. The texts that had been obtained through transcribed interviews and field records were broken down, and the recording units were regrouped according to the categories. The triangulation of the data^11^ that had been obtained from the interviews, from the field observations, and from the evidence that had been found in several secondary sources was used. The correlation analysis of cases was conducted as pointed out by Stake:^18^ by emphasizing the main findings in each case, comparing those findings among cases, and identifying factors to be analyzed.

This study was approved by the Research Ethics Committee of Escola Nacional de Saúde Pública (Official Opinion135.016). The Prison Administration Offices of both states formally approved the conduction of the research.

## RESULTS

The increased social vulnerability in case 2, with higher demographic densities and higher share of residents in subnormal clusters, may have contributed to the higher incidence of the disease in the prison system of that state (Table 1). TB incidence and Aids prevalence rates, which were higher in case 2, increased susceptibility to transmission and sickness.

**Table 1:** Cross analysis of cases: External dimension (adapted from Stake18). Brazil, 2014.

Sub-dimensions	Factors	Case 1	Case 2
Sociodemographic vulnerability to tuberculosis infection and sickening	Life conditions	Unfavorable: High demographic density, share of residents in subnormal communities similar to the national rate.	Very unfavorable: High demographic density and share of residents in subnormal communities.^a^
	Incidence of tuberculosis and prevalence of HIV/Aids	Unfavorable: Incidence of tuberculosis that is a little higher than the national rate in the general population and lower in the incarcerated population. High HIV/Aids prevalence.	Very unfavorable: Incidence of tuberculosis that is a little higher than the national rate both in the general population and in the incarcerated population. High HIV/Aids prevalence.
	Relationship between average monthly earnings^b^	Favorable: 11.9 Below the national average.	Unfavorable:15.2 Similar to the national average.

Political commitment	State funds applied in health care	Favorable: 11.0%	Favorable: 12.0%
	Funds from the Prison Administration applied in health care	1.7%^c^	1.0%^c^
	Excess population in the state prison system	Very unfavorable: 47.7%	Unfavorable: 28.4%

a Census sectors located in slums, invaded areas, grottoes, valleys, communities, villages, coastal shacks, huts, stilt constructions, among other irregular settlements.

b Ratio (A/B) between per capita average earnings from families in the 20.0% wealthiest group (A) and the 20.0% poorest group (B) in the state.

c The State Prison Administration Office is scheduled to take part in the financing of health care actions in the prison system, but the share or amount of funds to be applied has not been established.

The low use of the financial resources that had been on-lent by the Ministries of Health and Justice, and the failure to send the Annual Management Report caused the transference of funds for both cases to be canceled.

An external dimension that was unfavorable for TB control was observed in both cases, with a special mention to case 2 due to its epidemiological status, due to the life conditions from part of the population favoring transmission, and due to the insufficient financial investment for actions to be conducted. Overcrowded prisons with poor environmental conditions constituted a barrier in the fight against the disease in both cases.

The organization models for prison health care actions and TCP were distinct in both cases (Table 2). In case 1, with the large number of prisons spread all over the state, prison TCP management used to be part of Prison Health Care Coordination and TCP State Coordination. In case 2, with prisons concentrated in the metropolitan region, a qualified prison health care professional was in charge of the management. That professional organized professional training, the search for cases, the information system, and the supervision of the self-administered treatment of cases by a specific team.

**Table 2:** Cross analysis of cases: political-organizational dimension (adapted from Stake18). Brazil, 2014.

Sub-dimensions	Factors	Case 1	Case 2
Technical and political-financial autonomy	Assured infrastructure, trained professionals, and sufficient material resources for the actions	Unfavorable: Difficulty executing financial resources. Unmotivated health care team. Insufficient material resources.	Unfavorable: Difficulty executing financial resources. Unmotivated health care team. Insufficient material resources.
	Supply of medications	Favorable: Proper for tuberculosis-specific medications.	Favorable: Proper for tuberculosis-specific medications.
	Participation in planning and decision on resources for prison health care	Unfavorable: Small participation from health care managers and professionals.	Unfavorable: Small participation from health care managers and professionals.

Intersectorial actions	Assured transportation and escorting to reference units	Unfavorable: A vehicle that is not exclusive to health care, prioritizing other actions.	Unfavorable: A vehicle that is not exclusive to health care, prioritizing other actions.
	Health care team previously informed of transfers and releases	Unfavorable: Communication with little notice.	Very unfavorable: Lack of communication for most times.

Management	Integration between Prison Health Care and State/Municipal Health Care	Favorable: Well established in actions regarding training, supervision of results, and diagnostic support.	Unfavorable: Asystematic, restricted to specific contributions.
	Laboratory and radiological service support with enough funds to conduct indicated exams	Favorable: Decentralized flow. The health care units in prisons submit inmates to get exams done on laboratory networks in the municipalities or in the state.	Unfavorable: Centralized in the prison hospital. Inmates need to be transported. Laboratory and radiological exams ensured by the prison system. Lack of funds for HIV, culture, and AST tests for all cases.

PU: Prison units; AST: Antibiotic susceptibility testing

The offer of hospital beds for all kinds of admission types was 1 for 152 inmates in case 1, and 1 to each 46 inmates in case 2 (these data do not figure in the Tables). The prison hospitals were located in the metropolitan regions of both states. The prisons that were far away from the capital had access to the network of public and accredited hospitals in the municipalities from case 1.

Technical and political-financial autonomy were sub-par in both states, which hindered the use of funds from the financial incentive and the conduction of some of the proposed health care actions. The organizational structures of the prison systems of both states, in which health care professionals reported to prison management, restricted their participation in planning and organizing health care actions. The health care professionals indicated different players as the ones responsible for coordinating the actions. They almost never indicated the Prison System Health Care Coordination, which is considered by most as an agency which is not easily accessible in their everyday practice.

TB diagnose in case 1 involved treating inmates in the prison health care units, conducting laboratory exams in the municipal or state health care network, and submitting inmates to radiological exams. The directly observed treatment was established for diagnosed cases. In case 2, the flow involved forwarding inmates to prison hospitals, where clinical, bacilloscopy, and radiological exams were provided for all symptomatic cases. The treatment was self-administered and prescribed by specialist doctors.

As of 2007, incarceration conditions started figuring in the form for notifying TB cases in Brazil. The data from the records of analysis units 1 and 2 (of case 1) and the data from SINAN regarding the set of all prisons in each state were accessed. In case 2, the records were centralized in the prison hospital and no consolidated reports of cases in the prisons existed.

The results from TB treatments from 2007 to 2012, regarding all prisons from the states (SINAN), showed a large share of ignored outcomes in both cases (Tables 3 and 4). In 2011, there was a smaller share of ignored outcomes. In that year, cure and treatment abandonment rates were more favorable in prisons of case 1.

**Table 3:** Case 1: final status of tuberculosis cases in all prisons. Brazil, 2014.

Year of diagnose	Ignored/Blank		Cured		Treatment abandonment		Tuberculosis-related death		Death due to other causes		Transference		Total cases
	n	%	n	%	n	%	n	%	n	%	n	%	
2007	19	10.3	120	**65.2**	32	17.4	5	2.7	8	4.4	0	0	184
2008	69	7.4	731	**78.3**	102	10.9	3	0.3	27	2.9	2	0.2	934
2009	507	30.6	1,015	**61.2**	96	5.8	11	0.6	23	1.4	7	0.4	1,659
2010	1,180	**71.7**	391	23.7	35	2.1	4	0.2	36	2.2	1	0.1	1,647
2011	113	5.8	1,665	**85.2**	124	**6.3**	10	0.5	36	1.8	7	0.4	1,955
2012	619	29.9	1,305	**62.9**	110	5.3	8	0.4	26	1.3	5	0.2	2,073
Total	2,507	29.7	5,227	61.8	499	5.9	41	0.5	156	1.9	22	0.3	8,452

Source: Information System on Diseases of Compulsory Declaration (SINAN) (cited 2014 Feb 27).

In bold face, more favorable cure ratios than the ones from case 2, especially in the year with the smallest rate of ignored outcomes (2011), also with a more favorable treatment abandonment rate.

**Table 4:** Case 2: final status of tuberculosis (TB) cases in all prisons. Brazil, 2014.

Year of diagnose	Ignored/Blank		Cured		Treatment abandonment		Tuberculosis-related death		Death due to other causes		Transference		MDR		Total cases
	n	%	n	%	n	%	n	%	n	%	n	%	n	%	
2007	531	**72.1**	136	18.5	38	5.2	9	1.2	3	0.4	19	2.6	0	0	736
2008	720	**85.6**	77	9.1	17	2.0	11	1.3	3	0.4	13	1.5	1	0.1	842
2009	693	**84.2**	76	9.2	24	2.9	6	0.7	6	0.7	18	2.2	1	0.1	824
2010	615	**93.9**	16	2.4	8	1.2	2	0.3	5	0.8	9	1.4	0	0	655
2011	64	7.9	506	**62.7**	91	**11.3**	11	1.4	18	2.2	116	14.4	1	0.1	807
2012	298	**44.4**	249	37.1	57	8.5	15	2.2	8	1.2	42	6.3	2	0.3	671
Total	2,921	64.4	1,060	23.4	235	5.2	54	1.2	43	0.9	217	4.8	5	0.1	4,535

Source: Information System on Diseases of Compulsory Declaration (SINAN) (cited 2014 Feb 27).

MDR: Multiple drug resistance

In bold face, a large share of ignored outcomes; in 2011, the cure and treatment abandonment rates were less favorable than in case 1.

The incidence rate of TB in the prison system remained stable in case 1 (around 1,000/100,000). In case 2, a reduction in 2012 happened (from around 2,700 cases to 1,984/100,000 – data not figuring in the Tables).

Most of the inmates being treated for TB were found to be satisfied with the service in prison health care units when interviewed. However, they complained about the lack of physicians in the analysis units 2 and 5. The quality of food was criticized by the inmates from all prisons, except for the prison hospital (analysis unit 3). In case 2, the bad transportation conditions to have exams done led inmates to decline them, especially when they felt better. Overcrowded spaces and the large tobacco consumption in cells were considered to be harmful to treatment by the inmates in case 1.

The health care professionals considered confinement conditions and the lack of resources as obstacles for controlling TB in the prisons. Among the guards, there were frequent misconceptions regarding the inmates’ right to health care, as access to the services was seen as a privilege.

## DISCUSSION

The individual and social aspects that lead to increased or decreased susceptibility to infection and sickening, such as material conditions, social support, employment and earnings, among other factors, were described by Ayres et al.^2^ Those authors added a program-related dimension to vulnerability, and they consider the institutions as mediators between the subjects and their social contexts.^2^ Monteiro^12^ points out social inequalities that are present in the capitalist society and reinforces the need for structural changes. The incarcerated population mostly comprises individuals from structurally-deficient areas with difficult access to services, where tuberculosis prevalence is high. The relationships that are established among inmates, professionals, visitors, support groups, and, later, among freed individuals and their communities may favor TB spread in and out of prisons.

Case 2 kept the highest TB incidence rate in the country until 2012^g^ and high HIV/Aids rates in the general population of the state for many years,^5^ as well as a high share of residents in sub-normal communities.^6^ That favors higher TB incidence in the incarcerated population. Continuous planning, management, and funding for prison health care actions are key to control the disease. However, a low amount of resources was observed to be destined to sanitary needs, managers and health care professionals had little participation in the decisions regarding the application of funds and the continuous growth in the number of inmates. Independence of health care professionals in the prison system favors quality care.^1^ Nonetheless, the poor working conditions in prisons, the social and administrative disregard, and the low wages contribute to the lack of those professionals in the prison system and to the demotivation of many active professionals.^1^

The management autonomy is influenced by the financial autonomy, as the release of funds is key to financially support the services and to maintain the health care units. Centralized planning and decision regarding the use of resources limited the technical autonomy, which made the conduction of some planed actions impossible. In case 2, some TB patients had trouble getting laboratory exams, and there were difficulties regarding the extension of radiological monitoring and frequent supervision of patients in the prisons. Those actions depended on professionals and vehicles for transportation, but not always were they available. Thus, the political-organizational dimension has not favored the full implementation of the program. Those aspects were found in other studies for TCP evaluation.^13^ ^,^ ^15^

There are no validated standards for comparison analyses of the number of hospital beds per inhabitant, as that indicator expresses a combination of factors that is inherent to distinct regional or local realities.^h^ Hospital admission is only recommended by the Brazilian National Tuberculosis Control Program for special TB cases, or for inmates in prisons or police stations which do not have health care units.

The importance of external funds and agreements between prison administrations and external health care systems is highlighted by Arroyo and Astier,^1^ who suggest unified documentation that makes the exchange of information between prisons and external services easier, which would support ongoing care.

The recent implementation of Brazilian National Policy for Full Health Care of Inmates in the Prison System, through Interministry Directive 1/2014,^c^ proposes municipalized management, multidisciplinary teams, focus on the promotion of health and prevention of illnesses, which should be structured within the Health Care Networks. This policy matches suggestions that some authors^1^ ^,^ ^9^ present as possible solutions for the scarcity of and lack of motivation from health care professionals in the prison system: the integration of prison health care in the public health care system, with professionals providing care in and outside prisons, periodical work meetings, and networks for exchange of information among professionals. Zulaika et al^20^ argue that the responsibility by the health of inmates and the organization of health care in prisons should be transferred to the Health Care System, thus reducing dependency from health care professionals on prisons managers. The same authors inform that some countries, such as England and Scotland, took measures for their Ministries of Health to be responsible for health care services, instead of their Ministries of Justice.^20^

The context factors may either make it easy or difficult to execute the activities and reach intended results.^8^ The implementation of actions to control TB in the prisons, as standardized by the Brazilian National Prison System Health Care Plan and the Brazilian National Tuberculosis Control Program, take place in an unwelcoming environment, where the rights that are assured by the Sentence Execution Act are not actually ensured. Thus, the partial implementation that was reached by the prisons of case 1 and by the prison hospital of case 2 somehow represents success.

The theoretical model used assumes that multiple factors interact in the production of effects. Although the data regarding treatment outcomes are poor, the external and political-organizational dimensions and the partial implementation of TCP in the prisons of case 1 may influence cure and treatment abandonment rates in prison all over the state, especially considering the year with the smallest proportion of ignored outcomes. Conversely, the external and political-organizational dimensions and the low implementation degree of TCP in the prisons of case 2 may have influenced the less favorable results from the prisons in the state.

The cure rates of bacillary cases increased in the general populations of the two states (80.4% in case 1 and 64.0% in case 2, in 2010).^4^ That shows similarity with the data from 2011 regarding the prison population. The treatment abandonment rates for the general population were 9.7% and 12.0% in the states of cases 1 and 2 in 2010,^4^ which were worse than the ones observed for the incarcerated population. The reduced TB incidence in the incarcerated population from case 2 may arise from errors in the information system or due to underdetection.

The information on treatment outcomes was poor in both cases. The epidemiological data from the prisons were not provided by the Prison Administration Offices. That limited the analysis of the influence from studied dimensions on the program effects.

The compliance between theoretical propositions and the observed reality allowed validating the theoretical model of the adopted program. The triangulation of information from different sources, considering the similarities among them, has strengthened the internal validity of the study. The conduction of a multiple case study supported the external validity.

The study contributes to the construction of a model for evaluating the implementation of TB control actions in the prison system. The participation from stakeholders in the first phase of the study was fundamental for including practical knowledge in the developed models. The collection of data enabled mobilizing the players in the program and the reflection on the topic.

The problems which were detected in the prison TCP do not prevent it from performing its role in reducing morbidity and mortality rates due to TB or from its mitigating risk situations in the prison system. Public policies are indispensable for ensuring access to services. The Ministries of Health and Justice and the State Prison and Health Care Administration Offices must join efforts to implement policies that promote health in the Brazilian prison system. That would contribute to the health care services in the country.

## Footnotes

a Ministério da Saúde, Secretaria de Atenção em Saúde, Departamento de Ações Programáticas Estratégicas. Legislação em saúde no sistema penitenciário. Brasília (DF); 2010 [cited 2015 Apr 26]. Plano Nacional de Saúde no Sistema Penitenciário; p.128-45. Available from: http://bvsms.saude.gov.br/bvs/publicacoes/legislacao_saude_sistema_penitenciario.pdf

b Santos M, França P, Sanchez A, Larouzé B. Manual de intervenções ambientais para o controle da tuberculose nas prisões. Rio de Janeiro (RJ): Departamento Penitenciário Nacional; 2012 [cited 2015 Apr 26]. Available from: http://bvsms.saude.gov.br/bvs/publicacoes/manual_intervencoes_ambientais_controle_tuberculose_prisoes.pdf

c Ministério da Saúde; Ministério da Justiça. Portaria Interministerial n° 1, de 2 de janeiro de 2014. Institui a Política Nacional de Atenção Integral à Saúde das Pessoas Privadas de Liberdade no Sistema Prisional (PNAISP) no âmbito do Sistema Único de Saúde. Brasília (DF); 2014 [cited 2015 Apr 26]. Available from: http://bvsms.saude.gov.br/bvs/saudelegis/gm/2014/pri0001_02_01_2014.html

d Ministério da Saúde. Secretaria de Vigilância em Saúde. Tuberculose. Dados epidemiológicos. Brasília (DF), 2014 [cited 2015 Apr 26]. Available from: http://portalsaude.saude.gov.br/index.php/o-ministerio/principal/leia-mais-o-ministerio/741-secretaria-svs/vigilancia-de-a-a-z/tuberculose/11485-situacao-epidemiologica-dados

e Ministério da Justiça. Departamento Penitenciário Nacional. Infopen Sistema Integrado de Informações Penitenciárias. Brasília (DF), 2012 [cited 2015 Apr 26]. Available from: http://portal.mj.gov.br/data/Pages/MJD574E9CEITEMIDC37B2AE94C6840068B1624D28407509CPTBRIE.htm

f Ministério da Saúde. Secretaria de Atenção à Saúde. Datasus. CNESnet. Brasília (DF), 2014 [cited 2014 Feb 12]. Available from: http://cnes.datasus.gov.br/

g Ministério da Saúde. Série histórica da Taxa de Incidência de Tuberculose-Brasil, Regiões e Unidades Federadas -1990 a 2014. Brasília (DF); 2015 [cited 2015 Apr 26]. Available from: http://u.saude.gov.br/images/pdf/2014/abril/09/taxa-incidencia-tuberculose-1990-2012-base-DEZ-2013.pdf

h Instituto Brasileiro de Geografia e Estatística. Pesquisa de Assistência Médico-Sanitária (AMS) e base demográfica. Rio de Janeiro (RJ): IBGE; 2008.
