# Supplementary material for: Implementation contexts of a Tuberculosis Control Program in Brazilian prisons
Source: Rev Saude Publica. 2015 Sep 23;49:66. doi: 10.1590/S0034-8910.2015049005802 (PMC4587822; doi:10.1590/S0034-8910.2015049005802)
Supplement: Translation

# Translation: Contextos de implantação do Programa de Controle da Tuberculose nas prisões brasileiras

**Authors:** Luisa Gonçalves Dutra de Oliveira, Sonia Natal, Luiz Antonio Bastos Camacho

**Affiliations:** I Departamento de Planejamento em Saúde. Instituto de Saúde Coletiva. Universidade Federal Fluminense. Niterói, RJ, Brasil; II Departamento de Saúde Pública. Universidade Federal de Santa Catarina. Florianópolis, SC, Brasil; III Departamento de Epidemiologia e Métodos Quantitativos em Saúde. Escola Nacional de Saúde Pública Sérgio Arouca. Fundação Oswaldo Cruz. Rio de Janeiro, RJ, Brasil

**Keywords:** Prisioneiros, Prisões, Tuberculose, prevenção & controle, Avaliação de Programas e Projetos de Saúde, Pesquisa Qualitativa

## Abstract

**OBJETIVO:**

Analisar a influência das características contextuais do controle da tuberculose nas prisões e a influência dos graus de implantação do programa nos efeitos observados.

**MÉTODOS:**

Estudo de casos múltiplos, com abordagem qualitativa, realizado no sistema penitenciário de dois estados brasileiros em 2011 e 2012. As unidades de análise foram duas unidades prisionais em cada estado e um hospital penal em um deles. Os dados foram objeto de análise de conteúdo, que teve como eixo condutor as dimensões externa, político-organizacional, implantação e efeitos. Foi realizada correlação entre os fatores contextuais e da organização do programa que influenciaram a implantação das ações. A variável independente foi o grau de implantação do programa e a dependente, os efeitos do Programa de Controle da Tuberculose nas unidades prisionais.

**RESULTADOS:**

O contexto com maior vulnerabilidade sociodemográfica, maior taxa de incidência de tuberculose e menor disponibilidade de recursos associou-se ao baixo nível de implantação do programa. Os desfechos do tratamento da tuberculose no sistema prisional, nos dois casos, foram melhores onde o grau de implantação verificado foi parcialmente implantado, do que no caso com baixo nível de implantação.

**CONCLUSÕES:**

O grau de implantação e os contextos – dimensões externa e político-organizacional – contribuem simultaneamente para os efeitos observados no controle da tuberculose nas prisões analisadas.

## INTRODUÇÃO

As ações de controle da tuberculose (TB) no sistema prisional brasileiro foram normatizadas pelo Plano Nacional de Saúde do Sistema Penitenciário, em 2003, e das recomendações do Programa Nacional de Controle de Tuberculose, em 2010. As metas pactuadas foram: cura de pelo menos 85,0%, visando reduzir a transmissão e a ocorrência de casos novos, e diminuição do abandono a menos de 5,0%, com melhor prognóstico e menor risco de ocorrência de resistência às drogas de primeira linha.

Cada unidade federativa planeja e executa seu Plano Operativo Estadual (POE) de Saúde Penitenciária. Pode haver formas distintas de organização e gerenciamento das ações. A integração entre as instâncias da administração penitenciária e da saúde nos âmbitos federal, estaduais e municipais é fundamental para o desenvolvimento das ações com protocolo estabelecido e por profissionais motivados e capacitados para promover o diagnóstico precoce e o tratamento exitoso.^17^ ^,^ ^a^ As Secretarias Municipais de Saúde dos municípios que sediam unidades prisionais (UP) contribuem com a realização de exames complementares, ações de maior complexidade e assistência hospitalar aos apenados. Além da abordagem biomédica, são necessárias medidas educativas e de melhoria das condições ambientais para o controle da endemia nas prisões.^b^

O financiamento dos programas de saúde penitenciária é compartilhado pelo Ministério da Justiça e Ministério da Saúde, com a participação das instâncias estaduais de administração penitenciária e saúde. O incentivo financeiro para atenção à saúde penitenciária, criado em 2003, destina-se à UP com mais de 100 pessoas presas e que tenha unidade de saúde própria, com equipe mínima.^c^

Há pouco conhecimento sobre os fatores que influenciam a implantação das ações do Programa de Controle de Tuberculose (PCT) no sistema prisional. Este estudo é uma das etapas da pesquisa “Avaliação da Implantação do PCT no Sistema Penitenciário Brasileiro”. A primeira etapa, estudo de avaliabilidade, constatou a existência de diferentes modelos de organização nos casos selecionados.^14^ Na segunda etapa, estudo de casos múltiplos,^11^ ^,^ ^19^ foram analisados os graus de implantação do programa em cinco UP de dois Sistemas Estaduais Penitenciários.^16^

Este estudo teve como objetivo analisar a influência das características contextuais do controle da tuberculose nas prisões e a influência dos graus de implantação do programa nos efeitos observados.

## MÉTODOS

Estudo de casos múltiplos,^10^ ^,^ ^11^ ^,^ ^19^ com abordagem qualitativa. Os critérios de seleção dos casos foram: existência do PCT implantado, com tratamento ambulatorial ou hospitalar próprios, e existência de grande número de casos novos de TB registrados. Os casos selecionados, um com a maior incidência de TB no sistema prisional e outro com a maior população carcerária no País, tinham diferentes contextos e formas de organização do programa.^14^ Foram consideradas somente unidades com detentos do sexo masculino, pela predominância de homens no sistema carcerário e prevalência mais acentuada de TB em homens, tanto livres quanto encarcerados. Os casos foram os Sistemas de Saúde Penitenciária de dois estados e as unidades de análise, duas UP de cada estado. Em um dos casos, foi incluído um hospital penal entre as unidades de análise, onde as ações eram centralizadas.

As dimensões utilizadas no modelo teórico do programa foram: externa, político-organizacional, implantação propriamente dita e efeitos. A dimensão externa, correspondente ao contexto estadual, teve como subdimensões a vulnerabilidade sociodemográfica e o compromisso político*. *Para a primeira, as categorias definidas foram os aspectos demográficos, a situação socioeconômica da população, a incidência da TB e a prevalência da aids, como fatores condicionantes da suscetibilidade da população à transmissão e adoecimento por tuberculose. A subdimensão compromisso político foi determinada pelo apoio logístico e material para as ações de saúde por parte das secretarias de saúde e secretarias de administração penitenciária.

As subdimensões da dimensão político-organizacional analisadas foram autonomia técnica e político-financeira, ações intersetoriais e gerência. Na primeira*, *as categorias gestão do POE e utilização do incentivo financeiro indicaram a participação dos gestores e dos profissionais de saúde no planejamento e na decisão sobre os recursos e ações do programa. As ações intersetoriais*, *integração entre as áreas de saúde e de segurança, pretendiam garantir a execução das ações de controle da TB nas UP. Na gerência, foram consideradas as formas de organização do PCT no sistema penitenciário dos estados e os fluxos estabelecidos para as ações de diagnóstico, acompanhamento e tratamento dos casos.

A dimensão implantação correspondeu à execução das ações de controle da TB como normatizado e a dimensão efeitos considerou os resultados operacionais: taxas de cura e letalidade, adesão ao tratamento e satisfação da população privada de liberdade com a atenção à saúde.

O Modelo Teórico do Programa utilizou a tipologia de análise de implantação proposta por Champagne et al^7^ (Figura): (1) Os determinantes contextuais externos e político-organizacionais podem influenciar de maneira sinérgica ou antagônica a implantação das ações de controle da TB nas UP; (2) O grau de implantação do programa influencia os efeitos atribuídos ao mesmo; (3) A interação entre as dimensões pode também influenciar os efeitos observados.

**Figura:** Modelo Teórico do Programa de Controle da Tuberculose (PCT) no sistema prisional. Brasil, 2012.

A etapa anterior deste estudo delimitou os graus de implantação do PCT nas diversas unidades de análise de acordo com os resultados obtidos para cinco categorias: acesso; infraestrutura; busca de casos; diagnóstico e acompanhamento; e tratamento e assistência farmacêutica. Foram estabelecidos pontos de corte de acordo com a proporção do somatório dos pontos nas categorias em relação à pontuação estimada. Foram constatados programas parcialmente implantados (51,0% a 75,0%) em três unidades de análise e com baixo nível de implantação (26,0% a 50,0%) em duas unidades de análise.^16^

Tomou-se como variável dependente o grau de implantação da intervenção para analisar a influência das dimensões externa e político-organizacional na implantação nos dois casos. A análise de como as diferenças no grau de implantação fizeram variar os efeitos atribuídos à intervenção teve como variável dependente o efeito postulado. Analisou-se se a interação entre a intervenção e o seu contexto influenciava os efeitos.

Os dados primários foram obtidos por meio de entrevistas semiestruturadas e observação em campo, utilizando roteiros previamente elaborados e registros em áudio e em caderno de campo. Foram entrevistados, como representantes da coordenação de saúde penitenciária, o diretor de ações de saúde e os diretores de saúde das duas UP selecionadas, no caso 1, e o responsável pelo PCT prisional e o diretor do hospital penal para tratamento de TB, no caso 2. Nas cinco unidades de análise, foram entrevistados 37 profissionais de saúde (médicos, enfermeiros, auxiliares de enfermagem, dentista e assistente social), 14 agentes de segurança selecionados em conjunto com o chefe de segurança, priorizando-se aqueles que trabalhavam junto às unidades de saúde, e 42 detentos em tratamento para TB.

Os dados secundários foram provenientes de documentos e normas do programa, legislação de saúde penitenciária, POE, informações fornecidas pelo Instituto Brasileiro de Geografia e Estatística, Sistema de Informação de Agravos de Notificação (SINAN),^d^ Sistema Integrado de Informações Penitenciárias,^e^ Relatórios de gestão e Cadastro Nacional de Estabelecimentos de Saúde.^f^ Os dados quantitativos foram apresentados em números absolutos e percentagens.

Análise de conteúdo^3^ foi utilizada para a análise dos dados qualitativos. Realizou-se o desmembramento dos textos obtidos pela transcrição das entrevistas e dos registros de campo e reagrupamento das unidades de registro de acordo com as categorias. Foi utilizada a triangulação entre os dados^11^ obtidos das entrevistas, das observações de campo e das evidências encontradas nas diversas fontes secundárias. A análise imbricada dos casos foi realizada a partir dos passos apontados por Stake:^18^ enfatizar os principais achados de cada caso, fazer o cruzamento desses achados entre os casos e identificar os fatores para análise.

Este estudo foi aprovado pelo Comitê de Ética em Pesquisa da Escola Nacional de Saúde Pública (Parecer 13.477/2012). As Secretarias Estaduais de Administração Penitenciária dos dois estados aprovaram formalmente a realização da pesquisa.

## RESULTADOS

A maior vulnerabilidade social do caso 2, com maiores densidade demográfica e proporção de residentes em aglomerados subnormais, pode ter contribuído para a incidência superior da doença no sistema penitenciário daquele estado (Tabela 1). As taxas de incidência de TB e prevalência de aids, mais altas no caso 2, aumentaram a suscetibilidade à transmissão e ao adoecimento.

**Tabela 1:** Análise cruzada dos casos: Dimensão Externa (adaptado de Stake18). Brasil, 2014.

Subdimensões	Fatores	Caso 1	Caso 2
Vulnerabilidade sociodemográfica à infecção e adoecimento por tuberculose	Condições de vida	Desfavorável: Alta densidade demográfica, proporção de residentes em aglomerados subnormais semelhante à taxa nacional.	Muito desfavorável: Alta densidade demográfica e alta proporção de residentes em aglomerados subnormais.^a^
	Incidência de tuberculose e prevalência de HIV/aids	Desfavorável: Incidência de tuberculose pouco maior que a nacional na população em geral e menor na população privada de liberdade. Alta prevalência de HIV/aids.	Muito desfavorável: Incidência de tuberculose maior que a nacional tanto na população em geral como na população privada de liberdade. Alta prevalência de HIV/aids.
	Relação entre rendimento médio mensal^b^	Favorável: 11,9 Abaixo da média nacional.	Desfavorável: 15,2 Semelhante à média nacional.
Compromisso político	Recursos estaduais aplicados na saúde	Favorável: 11,0%	Favorável: 12,0%
	Recursos da administração penitenciária aplicados na saúde	1,7%^c^	1,0%^c^
	Excedente populacional no sistema penitenciário estadual	Muito desfavorável: 47,7%	Desfavorável: 28,4%

a Setores censitários localizados em favelas, invasões, grotas, baixadas, comunidades, vilas, ressacas, mocambos, palafitas, entre outros assentamentos irregulares.

b Relação (A/B) entre o rendimento médio mensal familiar *per capita* das famílias com rendimento dos 20,0% mais ricos (A) e dos 20,0% mais pobres (B) no estado.

c Está prevista a participação da Secretaria Estadual de Administração Penitenciária no financiamento das ações de saúde no Sistema Penitenciário, mas não estabelece-se a proporção ou o volume de recursos a ser aplicado.

A baixa utilização do recurso do incentivo financeiro repassado pelos Ministérios da Saúde e da Justiça e o não envio do Relatório Anual de Gestão resultaram na suspensão do repasse para ambos os casos.

Foi observada dimensão externa desfavorável ao controle da TB em ambos os casos, com destaque para o caso 2 por sua situação epidemiológica, pelas condições de vida de parte da população propícias à transmissão, e pelo investimento financeiro insuficiente para a realização das ações. A superlotação das UP, com condições ambientais precárias, constituiu barreira no controle da doença nos dois casos.

As formas de organização das ações de saúde penitenciária e do PCT eram distintas nos dois casos (Tabela 2). No caso 1, com grande número de UP dispersas por todas as regiões do estado, o gerenciamento do PCT prisional integrava a Coordenação de Saúde Penitenciária e a Coordenação Estadual do PCT. No caso 2, com UP concentradas na região metropolitana, a gerência cabia a um profissional qualificado da saúde penitenciária. Esse profissional organizava a capacitação de recursos humanos, a busca de casos, o sistema de informação e o acompanhamento do tratamento autoadministrado dos casos por equipe própria.

**Tabela 2:** Análise cruzada dos casos: dimensão político-organizacional (adaptado de Stake18). Brasil, 2014.

Subdimensões	Fatores	caso 1	caso 2
Autonomia técnica e político-financeira	Garantia de infraestrutura, recursos humanos capacitados e recursos materiais suficientes para as ações	Desfavorável: Dificuldade na execução dos recursos financeiros. Equipe de saúde desmotivada. Recursos materiais insuficientes.	Desfavorável: Dificuldade na execução dos recursos financeiros. Equipe de saúde desmotivada. Recursos materiais insuficientes.
	Suprimento de medicamentos	Favorável: Adequado para os medicamentos específicos para tuberculose.	Favorável: Adequado para os medicamentos específicos para tuberculose.
	Participação no planejamento e decisão sobre os recursos para a saúde penitenciária	Desfavorável: Participação incipiente dos gerentes e profissionais de saúde.	Desfavorável: Participação incipiente dos gerentes e profissionais de saúde.
Ações intersetoriais	Garantia de transporte e escolta para as referências	Desfavorável: Veículo não exclusivo da área de saúde, privilegiando outras ações.	Desfavorável: Veículo não exclusivo da área de saúde, privilegiando outras ações.
	Transferências e libertações comunicadas à equipe de saúde com antecedência	Desfavorável: Comunicação feita com pouca antecedência.	Muito desfavorável: Falta de comunicação na maior parte das vezes.
Gerência	Integração entre saúde penitenciária e saúde estadual/municipal	Favorável: Bem estabelecida nas ações de capacitação, monitoramento dos resultados e apoio diagnóstico.	Desfavorável: Assistemática, restrita a colaborações pontuais.
	Suporte de serviço laboratorial e radiológico com recursos suficientes para realização dos exames indicados	Favorável: Fluxo descentralizado. As unidades de saúde das UP encaminham os exames para a rede laboratorial dos municípios-sede ou estado.	Desfavorável: Centralizado no hospital penal. Necessário o deslocamento do detento. Garantia de exame laboratorial e radiológico pelo sistema penitenciário. Carência de recursos para exames anti-HIV, cultura e TSA para todos os casos.

UP: unidades prisionais; TSA: teste de sensibilidade aos antimicrobianos

A oferta de vagas hospitalares para todos os tipos de internação foi de uma para 152 no caso 1, e de uma para 46 no caso 2 (dados não apresentados em Tabelas) em relação ao total de detentos. Os hospitais penitenciários estavam localizados nas regiões metropolitanas de ambos os estados. As prisões distantes da capital contavam com a rede de hospitais públicos e conveniados dos municípios-sede no caso 1.

A autonomia técnica e político-financeira foi deficiente nos dois estados, o que dificultou a utilização dos recursos do incentivo financeiro e a realização de parte das ações de saúde propostas. A estrutura organizacional do sistema penitenciário dos dois estados, em que os profissionais de saúde estavam hierarquicamente subordinados à direção da UP, restringiu a sua participação no planejamento e na organização das ações de saúde. Os profissionais de saúde identificavam diferentes atores como responsáveis pela coordenação das ações. Quase nunca indicavam a Coordenação de Saúde do Sistema Penitenciário, considerada pela maioria como um órgão distante da sua prática cotidiana.

O diagnóstico de TB no caso 1 envolvia o atendimento ao detento na unidade de saúde da UP, realização dos exames laboratoriais na rede municipal ou estadual e encaminhamento ao exame radiológico. O tratamento diretamente observado era instituído para os casos diagnosticados. No caso 2, o fluxo envolvia, além do atendimento na unidade de saúde da UP, o encaminhamento ao hospital penal onde eram realizados os exames clínico, baciloscópico e radiológico para todos os sintomáticos. O tratamento era autoadministrado, prescrito pelo médico especialista.

A partir de 2007, a condição de encarceramento passou a constar na ficha de notificação dos casos de TB no Brasil. Foram acessados os dados dos livros de registro das unidades de análise 1 e 2, do caso 1, e os dados do SINAN relativos ao conjunto de todas as UP de cada estado. No caso 2, os registros eram centralizados no hospital penal e não havia um consolidado do monitoramento dos casos nas UP.

Os resultados dos tratamentos de TB de 2007 a 2012, relativos a todas as UP dos estados (SINAN), mostraram grande proporção de desfechos ignorados em ambos os casos (Tabelas 3 e 4). Em 2011, houve menor proporção de desfechos ignorados e naquele ano as taxas de cura e de abandono de tratamento foram mais favoráveis nas UP do caso 1.

**Tabela 3:** Caso 1: situação de encerramento dos casos de tuberculose de todas as unidades prisionais. Brasil, 2014.

Ano de diagnóstico	Ignorado/Branco		Cura		Abandono		Óbito por tuberculose		Óbito por outras causas		Transferência		Total de casos
	n	%	n	%	n	%	n	%	n	%	n	%	
2007	19	10,3	120	**65,2**	32	17,4	5	2,7	8	4,4	0	0	184
2008	69	7,4	731	**78,3**	102	10,9	3	0,3	27	2,9	2	0,2	934
2009	507	30,6	1.015	**61,2**	96	5,8	11	0,6	23	1,4	7	0,4	1.659
2010	1.180	**71,7**	391	23,7	35	2,1	4	0,2	36	2,2	1	0,1	1.647
2011	113	5,8	1.665	**85,2**	124	**6,3**	10	0,5	36	1,8	7	0,4	1.955
2012	619	29,9	1.305	**62,9**	110	5,3	8	0,4	26	1,3	5	0,2	2.073
Total	2.507	29,7	5.227	61,8	499	5,9	41	0,5	156	1,9	22	0,3	8.452

Fonte: Sistema Nacional de Agravos de Notificação (citado 2014 fev 27).

Em negrito, proporções de cura mais favoráveis que as do caso 2, especialmente no ano com menor taxa de desfechos ignorados (2011), com taxa de abandono também mais favorável.

**Tabela 4:** Caso 2: situação de encerramento dos casos de tuberculose (TB) de todas as unidades prisionais. Brasil, 2014.

Ano de diagnóstico	Ignorado/Branco		Cura		Abandono		Óbito por tuberculose		Óbito por outras causas		Transferência		MDR		Total de casos
	n	%	n	%	n	%	n	%	n	%	n	%	n	%	
2007	531	**72,1**	136	18,5	38	5,2	9	1,2	3	0,4	19	2,6	0	0	736
2008	720	**85,6**	77	9,1	17	2,0	11	1,3	3	0,4	13	1,5	1	0,1	842
2009	693	**84,2**	76	9,2	24	2,9	6	0,7	6	0,7	18	2,2	1	0,1	824
2010	615	**93,9**	16	2,4	8	1,2	2	0,3	5	0,8	9	1,4	0	0	655
2011	64	7,9	506	**62,7**	91	**11,3**	11	1,4	18	2,2	116	14,4	1	0,1	807
2012	298	**44,4**	249	37,1	57	8,5	15	2,2	8	1,2	42	6,3	2	0,3	671
Total	2.921	64,4	1.060	23,4	235	5,2	54	1,2	43	0,9	217	4,8	5	0,1	4.535

Fonte: Sistema Nacional de Agravos de Notificação (citado 2014 fev 27).

MDR: multi-droga resistente

Em negrito, grande proporção de desfechos ignorados e, em 2011, proporção de cura e de abandono foram menos favoráveis que no caso 1.

A taxa de incidência de TB no sistema prisional manteve-se estável no caso 1 (cerca de 1.000/100.000). No caso 2, houve redução em 2012 (de cerca de 2.700 casos para 1.984/100.000 – dados não apresentados em Tabelas).

A maior parte dos detentos em tratamento para TB entrevistados mostrou-se satisfeita com o atendimento dos serviços de saúde nas prisões. Entretanto, os usuários queixaram-se da carência de médicos nas unidades de análise 2 e 5. A qualidade da alimentação foi criticada pelos detentos em todas as UP, com exceção do hospital penal (unidade de análise 3). No caso 2, as más condições do transporte para realização de exames levavam detentos a abdicarem deles, especialmente quando se sentiam em melhor estado. A superlotação e o grande consumo de tabaco dentro da cela foram considerados prejudiciais ao tratamento pelos internos do caso 1.

Os profissionais de saúde consideraram as condições de confinamento e a carência de recursos como obstáculos ao controle da TB nas prisões. Entre os agentes de segurança, foi frequente a inconformidade com o direito do preso à assistência à saúde e a percepção de que o acesso aos serviços era um privilégio.

## DISCUSSÃO

Os aspectos individuais e sociais que acarretam maior ou menor suscetibilidade à infecção e ao adoecimento, como as condições materiais, suporte social, emprego e salário, entre outros, foram descritos por Ayres et al.^2^ Esses autores acrescentam uma dimensão programática à vulnerabilidade e consideram as instituições como mediadoras entre os sujeitos e seus contextos sociais.^2^ Monteiro^12^ aponta desigualdades sociais presentes na sociedade capitalista e reforça a necessidade de mudanças estruturais. A população carcerária, em sua maioria, é constituída de indivíduos provenientes de áreas com deficiências estruturais, dificuldade de acesso a serviços e onde a prevalência de tuberculose é elevada. As relações que se estabelecem entre presos, profissionais, visitantes, grupos de apoio e, posteriormente, entre internos, quando libertados, e suas comunidades, podem favorecer a disseminação da TB para dentro e para fora das prisões.

O caso 2 manteve até 2012 a maior incidência de TB no País^g^ e taxas elevadas de HIV/aids na população geral do estado por muitos anos,^5^ bem como alta proporção de residentes em aglomerados subnormais.^6^ Isso favorece a maior incidência de TB na população encarcerada. O planejamento, gerência e financiamento contínuo para as ações de saúde prisional são essenciais para o controle da endemia. Observou-se, porém, baixa proporção de recursos destinados às necessidades sanitárias, pequena participação dos gerentes e profissionais de saúde nas decisões sobre a aplicação dos recursos e o crescimento contínuo do número de presos. A independência dos profissionais de saúde dentro da administração penitenciária favorece a qualidade da assistência.^1^ Porém, as más condições de trabalho nas prisões, a desvalorização social e administrativa e a baixa remuneração contribuem para a falta desses profissionais no sistema prisional e para a desmotivação de muitos profissionais em atuação.^1^

A autonomia gerencial é influenciada pela autonomia financeira, visto que a liberação dos recursos é imprescindível para o custeio da assistência prestada aos usuários e para a manutenção das unidades de saúde. A centralização do planejamento e decisão quanto à utilização dos recursos limitou a autonomia técnica, o que impossibilitou a realização de algumas ações planejadas. No caso 2, houve dificuldade em realizar exames laboratoriais previstos para os pacientes com TB, bem como a ampliação do rastreamento radiológico e o acompanhamento frequente dos pacientes em tratamento nas UP. Essas ações dependiam de profissionais e de veículo para transporte, nem sempre disponíveis. Dessa forma, a dimensão político-organizacional não favoreceu a plena implantação do programa. Esses aspectos foram encontrados em outros estudos de avaliação do PCT.^13^ ^,^ ^15^

Não há padrões validados para análises comparativas do número de leitos hospitalares por habitante, pois o indicador expressa uma combinação de fatores inerentes a realidades regionais ou locais distintas.^h^ A hospitalização para os casos de TB é recomendada pelo Programa Nacional de Controle de Tuberculose somente em casos especiais, ou para os detentos que se encontram em UP ou delegacias sem unidade de saúde.

A importância dos recursos externos e dos acordos entre a administração penitenciária e o sistema de saúde externo é destacada por Arroyo e Astier,^1^ que sugerem documentação unificada que facilite o intercâmbio de informações entre prisões e destas com os serviços externos, o que contribuiria para a continuidade da atenção.

A recente instituição da Política Nacional de Atenção Integral à Saúde das Pessoas Privadas de Liberdade no Sistema Prisional, por meio da Portaria Interministerial 1/2014,^c^ propõe gestão municipalizada, equipe multidisciplinar, foco na promoção da saúde e prevenção de agravos, estruturada dentro das Redes de Atenção à Saúde. Esta Política vai ao encontro de sugestões que autores^1^ ^,^ ^9^ apresentam como possíveis soluções para a escassez e desmotivação dos profissionais de saúde no sistema prisional: a integração da saúde penitenciária ao sistema público de saúde, com profissionais atendendo dentro e fora das prisões, reuniões de trabalho periódicas e rede de intercâmbio de informações entre profissionais. Zulaika et al^20^ defendem a transferência da responsabilidade pela saúde dos internos e pela organização assistencial nas prisões ao Sistema de Saúde, reduzindo a dependência dos profissionais de saúde aos diretores das UP. Os mesmos autores informam que alguns países, como Inglaterra e Escócia, mobilizaram-se para que os serviços de saúde sejam de responsabilidade do Ministério da Saúde e não mais do Ministério da Justiça.^20^

Os fatores contextuais podem constituir facilidades ou barreiras, contribuindo para o êxito ou dificultando a execução das atividades e o alcance dos resultados.^8^ A implantação das ações de controle da TB nas UP como normatizadas pelos Plano Nacional de Saúde do Sistema Penitenciário e Programa Nacional de Controle de Tuberculose ocorre em um ambiente inóspito, em que os direitos assegurados pela Lei de Execução Penal não estão de fato garantidos. Assim, a implantação parcial alcançada pelas UP do caso 1 e pelo hospital penal do caso 2 representam, de certa forma, um êxito.

O modelo teórico utilizado pressupõe que múltiplos fatores interagem na produção dos efeitos. Embora os dados relativos ao desfecho do tratamento sejam precários, as dimensões externa, político-organizacional e a implantação parcial do PCT nas UP do caso 1 podem influenciar as taxas de cura e de abandono nas prisões de todo o estado, considerando especialmente o ano com menor proporção de desfechos ignorados. Da mesma forma, as dimensões externa, político-organizacional e o baixo nível de implantação do PCT nas prisões do caso 2 podem ter influenciado os resultados menos favoráveis nas prisões do estado.

As taxas de cura dos casos bacilíferos aumentou na população em geral dos dois estados (80,4% no caso 1 e 64,0% no caso 2, em 2010).^4^ Isso mostra similaridade com os dados de 2011 da população carcerária. As taxas de abandono para a população em geral foram de 9,7% e 12,0% nos estados dos casos 1 e 2 em 2010,^4^ taxas piores dos que as obtidas pela população privada de liberdade. A redução na incidência de TB na população privada de liberdade do caso 2 pode decorrer de falhas no sistema de informação ou da subdetecção.

A informação sobre os desfechos de tratamento foi precária em ambos os casos. Não foram fornecidos os dados epidemiológicos das UP pelas Secretarias de Administração Penitenciária. Isso limitou a análise da influência das dimensões estudadas sobre os efeitos do programa.

A conformidade entre as proposições teóricas e a realidade observada permitiu validar o modelo teórico do programa adotado. A triangulação das informações de diferentes fontes, considerando as convergências entre elas, fortaleceu a validade interna do estudo. A realização de estudo de casos múltiplos apoiou a validade externa.

O estudo contribui para a construção de um modelo de avaliação da implantação das ações de controle da TB no sistema prisional. A participação dos interessados na primeira fase do estudo foi fundamental para a inserção dos conhecimentos práticos nos modelos desenvolvidos. A coleta de dados propiciou a mobilização dos atores do programa e a reflexão sobre o tema.

Os problemas detectados no PCT prisional não o impedem de exercer seu papel na redução da morbimortalidade por TB e diminuir as situações de risco no sistema prisional. As políticas públicas são indispensáveis para garantir o acesso aos serviços. É imprescindível a união de esforços entre os Ministérios da Justiça e da Saúde e as Secretarias Estaduais de Administração Penitenciária e de Saúde para a implantação de políticas que promovam a saúde no sistema penitenciário brasileiro. Isso contribuiria para a saúde pública do País.

## Footnotes

a Ministério da Saúde, Secretaria de Atenção em Saúde, Departamento de Ações Programáticas Estratégicas. Legislação em saúde no sistema penitenciário. Brasília (DF); 2010 [citado 2015 abr 26]. Plano Nacional de Saúde no Sistema Penitenciário; p.128-45. Disponível em: http://bvsms.saude.gov.br/bvs/publicacoes/legislacao_saude_sistema_penitenciario.pdf

b Santos M, França P, Sanchez A, Larouzé B. Manual de intervenções ambientais para o controle da tuberculose nas prisões. Rio de Janeiro (RJ): Departamento Penitenciário Nacional; 2012 [citado 2015 abr 26]. Disponível em: http://bvsms.saude.gov.br/bvs/publicacoes/manual_intervencoes_ambientais_controle_tuberculose_prisoes.pdf

c Ministério da Saúde; Ministério da Justiça. Portaria Interministerial n° 1, de 2 de janeiro de 2014. Institui a Política Nacional de Atenção Integral à Saúde das Pessoas Privadas de Liberdade no Sistema Prisional (PNAISP) no âmbito do Sistema Único de Saúde. Brasília (DF); 2014 [citado 2015 abr 26]. Disponível em: http://bvsms.saude.gov.br/bvs/saudelegis/gm/2014/pri0001_02_01_2014.html

d Ministério da Saúde. Secretaria de Vigilância em Saúde. Tuberculose. Dados epidemiológicos. Brasília (DF), 2014 [citado 2015 abr 26]. Disponível em: http://portalsaude.saude.gov.br/index.php/o-ministerio/principal/leia-mais-o-ministerio/741-secretaria-svs/vigilancia-de-a-a-z/tuberculose/11485-situacao-epidemiologica-dados

e Ministério da Justiça. Departamento Penitenciário Nacional. Infopen Sistema Integrado de Informações Penitenciárias. Brasília (DF), 2012 [citado 2015 abr 26]. Disponível em: http://portal.mj.gov.br/data/Pages/MJD574E9CEITEMIDC37B2AE94C6840068B1624D28407509CPTBRIE.htm

f Ministério da Saúde. Secretaria de Atenção à Saúde. Datasus. CNESnet. Brasília (DF), 2014 [citado 2014 fev 12]. Disponível em: http://cnes.datasus.gov.br/

g Ministério da Saúde. Série histórica da Taxa de Incidência de Tuberculose-Brasil, Regiões e Unidades Federadas -1990 a 2014. Brasília (DF); 2015 [citado 2015 abr 26]. Disponível em: http://u.saude.gov.br/images/pdf/2014/abril/09/taxa-incidencia-tuberculose-1990-2012-base-DEZ-2013.pdf

h Instituto Brasileiro de Geografia e Estatística. Pesquisa de Assistência Médico-Sanitária (AMS) e base demográfica. Rio de Janeiro (RJ): IBGE; 2008.

## DESTAQUES

A prevalência da tuberculose na população privada de liberdade no Brasil tem se mantido elevada nos últimos anos, ainda que as taxas de incidência na população em geral tenham diminuído. Este estudo avaliou a implantação do Programa de Controle da Tuberculose no sistema penitenciário de dois estados brasileiros visando fornecer subsídios para tomada de decisões quanto aos ajustes e reformulações necessários à melhoria do programa.

Este estudo identificou dois modelos diferentes de organização das ações de controle da tuberculose nos dois casos estudados. Na análise da implantação, verificou-se que o programa estava parcialmente implantado nas unidades prisionais estudadas do Caso 1 e no hospital penal (do Caso 2), enquanto que, nas unidades prisionais não hospitalares do Caso 2, o nível de implantação era baixo. As taxas de cura e de abandono do tratamento foram mais favoráveis no Caso 1.

Fatores dificultadores do acesso aos serviços de saúde foram: a decisão sobre o encaminhamento sob responsabilidade dos guardas ou de um grupo de presos e a necessidade de escolta e a escassez de recursos humanos, financeiros e materiais para o desenvolvimento das ações. Em ambos os casos houve dificuldade para execução dos recursos financeiros destinados às unidades de saúde do sistema prisional.

Ações que favorecem o êxito do programa: busca ativa no ingresso do detento e nos exames periódicos e de contatos; tratamento diretamente observado para todos; comunicação entre os profissionais de segurança e os de saúde nas unidades prisionais, entre unidades por ocasião das transferências, e entre o sistema penitenciário e o sistema de saúde nas situações de livramento, de forma a evitar a interrupção do tratamento dos pacientes.

Rita de Cássia Barradas Barata Editora Científica
